# Analysis of web design visual element attention based on user educational background

**DOI:** 10.1038/s41598-024-54444-8

**Published:** 2024-02-26

**Authors:** Haohua Qing, Roliana Ibrahim, Hui Wen Nies

**Affiliations:** 1 Guangzhou College of Technology and Business, Guangzhou, Guangdong China; 2 Department of Applied Computing and Artificial Intelligence, Faculty of Computing, Universiti Teknologi Malaysia, Johor Bahru, Johor Malaysia

**Keywords:** Big data, Visual elements, Web design, Graphic visual elements, Computer science, Information technology

## Abstract

The evolution of Internet technology has led to an increase in online users. This study focuses on the pivotal role of visual elements in web content conveyance and their impact on user browsing behavior. Therefore, the use of visual elements in web design based on big data has aroused widespread concern among web designers, they apply visual elements to their web design works to make the web more attractive. This study examines the composition and distribution characteristics of key visual elements identified through user behavior data in a big data environment and discusses the use of visual elements in web design in the era of network economy. In addition, this paper issued 200 questionnaires to investigate the degree of attention to visual elements in web pages for users of different occupations and different educational backgrounds. Our survey indicated that visual elements captured the attention of 41% of corporate employees, whereas a mere 1% of social welfare workers focused on web content; 36% of undergraduates pay attention to visual elements of web pages, but only 5% and 4% of postgraduates and doctoral degrees and above. Therefore, the visual elements of the designed web page need to conform to the user's cultural background and professional background.

## Introduction

Websites emerged during the continuous development of Internet technology and are the key to users’ online search. With the rapid increase in the number of Internet users, people use the internet more and more frequently^1^. More companies want to pass the company's products, service concepts and corporate culture to the public through web pages^2^. Web design includes many visual elements, such as the text content, image content, color matching, and panel design of the web page^3^. The role of visual elements in web design is mainly to beautify web pages^4^. It can incorporate different content into web design. It can help people quickly understand and analyze things, and it can also make web design more in line with the current aesthetics, in order to realize the communication of web page information^5,6^. Based on this, the web designer has properly managed the visual elements in the web page, and found that the increase in visual intensity between the user experience and the visual impact can attract users^7^. By adjusting the visual elements of the web interface, a user-guided effect is achieved^8,9^.

Web designers design content for different web pages, using navigation and advertising to attract users' attention^10^. According to Fitz's law, the size of the content in the web interface will affect the user's behavior^11^. This problem can be solved by shrinking the content of the web page^12^. Based on the analysis of the big data environment, a dynamic evaluation method can be designed in the web page according to the needs, which can be used in the teaching system. Through the attraction of the visual elements of the web page, students can evaluate their learning situation on the Internet during their spare time^13,14^.

This paper mainly studies the use of visual elements in web design under the big data environment. This paper mainly studies the use of visual elements in web design under the big data environment and it mainly focuses on the following parts: First, analyze the application principles of big data in web design, and propose the influence of internet technology on the use of web design; second, study the function of visual elements in web design, and the influence of text content, image content, color matching and panel design that make up visual elements on web design; third, analyze the existing problems of current web design from a contemporary aesthetic point of view; based on this, propose strategies for the application of visual elements in web design.

In this paper, we define “visual elements” as the fundamental components that constitute web page design, such as text content, image content, color matching, and panel design. “Web design” refers to the process of integrating these visual elements to convey information and facilitate user interaction. Specifically, “network visual elements” refer to visual components used in a web environment to attract and maintain user attention.

Before delving into the functions and impact of visual elements, it is crucial to clarify the theoretical foundation upon which our study rests. Our research is built upon the principles of cognitive psychology and visual communication theory, focusing on how users process visual information and the impact of visual elements on web browsing behavior. Specifically, we are guided by Donald Norman's theory of emotional design^15^ and recent research on user experience within big data environments^16^ in constructing our analytical framework and research methodology.

## Related works

### Overview of big data technology and web design

With the rapid development of social economy and internet economy, the number of netizens in our country is increasing, and more and more companies use internet technology^17,18^. The development of big data has closely integrated corporate information with big data analysis. Personalization technology is the key point of big data analysis. Companies apply personalized technology to corporate web pages and use it as a platform for corporate information display and user communication^19,20^. As one of the basic algorithms of big data, Pagerank function assigns a value to web pages in the Web, the higher the Pagerank value is, the more important it is, and the higher the probability of web-page quality^21^. The Pagerank function is defined as:

1 $$PR\left({p}_{i}\right)=\left(1-d\right)+d\times {\sum }_{{p}_{j}\in M({p}_{i})}\frac{PR\left({p}_{j}\right)}{L\left({p}_{j}\right)}$$

In the Pagerank function, $PR\left({p}_{i}\right)$ denotes the Pagerank of page ${p}_{i}$. The damping factor, represented by $d$, is typically set to 0.85. The function considers $M({p}_{i})$ as the set of pages linking to ${p}_{i}$ and $L\left({p}_{j}\right)$ as the count of outbound links on the page ${p}_{j}$.

Abstract the web page in the Web into a node, and abstract the entire Web into a guidance graph to get the corresponding transition matrix^22^, as shown below:

2 $$ M = \left| {\begin{array}{*{20}c} 0 & {{\raise0.7ex\hbox{$1$} \!\mathord{\left/ {\vphantom {1 2}}\right.\kern-0pt} \!\lower0.7ex\hbox{$2$}}} & {{\raise0.7ex\hbox{$1$} \!\mathord{\left/ {\vphantom {1 2}}\right.\kern-0pt} \!\lower0.7ex\hbox{$2$}}} \\ {{\raise0.7ex\hbox{$1$} \!\mathord{\left/ {\vphantom {1 2}}\right.\kern-0pt} \!\lower0.7ex\hbox{$2$}}} & 0 & {{\raise0.7ex\hbox{$1$} \!\mathord{\left/ {\vphantom {1 2}}\right.\kern-0pt} \!\lower0.7ex\hbox{$2$}}} \\ {{\raise0.7ex\hbox{$1$} \!\mathord{\left/ {\vphantom {1 2}}\right.\kern-0pt} \!\lower0.7ex\hbox{$2$}}} & {{\raise0.7ex\hbox{$1$} \!\mathord{\left/ {\vphantom {1 2}}\right.\kern-0pt} \!\lower0.7ex\hbox{$2$}}} & 0 \\ \end{array} } \right| $$

The value in row i and column j represents the probability of jumping to web page i from web page j. The rank value of the web page at the beginning is 1/N, here is 1/3, the probability distribution of the first time going online is M^1^v, the second time going online is M^2^v, and so on, the probability of going online for the i-th time is M^i^v, the greater the probability of a user going online, the more attractive the visual elements of the web-page are. Using the Pagerank function big data algorithm, it can count the probability of users browsing different visual elements such as text, images, and sounds. The statistical data is used to improve web design and make web pages meet the needs of different users.

### The main visual elements that make up web design

The web platform is the main carrier for the transmission of Internet information. The most important thing in web design is its functions and the visual effects it brings to users. In order to improve the visual effects of web design, we first need to analyze the basic visual elements that constitute web design^23^. Specifically, the following aspects are crucial:

1. The visual element of text content, which expresses the author’s thoughts and feelings through text. It accounts for a large proportion in web design and contains a large amount of information. In the process of web design, the font color of visual elements of text content, style, and size will affect the overall layout of the web design.
2. The visual elements of image content. Images are an indispensable part of web design. The visual elements of image content bring stronger visual impact to the viewer than the visual elements of text content. It can highlight the web page in the web design process the style and attract the attention of viewers.
3. Color matching with visual elements, it can reflect the overall beauty of the web page in the web design process, and the combination of color visual elements and image visual colors can better highlight the theme of web design.
4. The visual elements of panel design are very important to web design. A reasonable design of web page layout can better express the visual color of text content, visual elements of image content, and color matching visual elements. It needs to start from the user's point of view, and the design is in line with the modern human aesthetic web page layout can attract more viewers.

After detailing the main visual elements of web design, it is essential to consider how these elements interact with one another and how they are perceived as a unified whole by users in accordance with Gestalt psychology principles, rather than merely isolated parts. For instance, how the visual elements of text and images combine to produce a synergistic effect, how color matching affects the unity of the visual theme, and how the layout guides the user's visual flow. These factors collectively define the user’s visual experience and comprehension, and thus, their interrelations must be meticulously considered in the design process.

Furthermore, this study will also analyze the differences in interest towards visual element content among user groups with different educational backgrounds. We will investigate which specific contents better engage certain user demographics, thus providing a basis for personalized web design.

### Principles of using visual elements in web design

The use of visual elements in the web design process can transmit web information more directly and effectively to viewers^24^. As a means of web design to convey information to users, visual elements need to be clarified about the principles of using visual elements in the web design process^25^. There are several guiding principles for using visual elements in web design, such as the principles of visibility outlined by Andy et al.^26^, and the user interface design rules by Matera et al.^27^: One is that the visual elements must be able to attract the user's attention; the second is that the content of the web page needs to be understood by the viewer; the third is that the visual elements in web design must conform to the user’s reading habits; the fourth is that the visual elements can allow users to leave a deep image and guide users to read; the fifth is to design personalized reading services that are convenient for viewers to browse according to user needs.

## Methods

**Figure 1:**
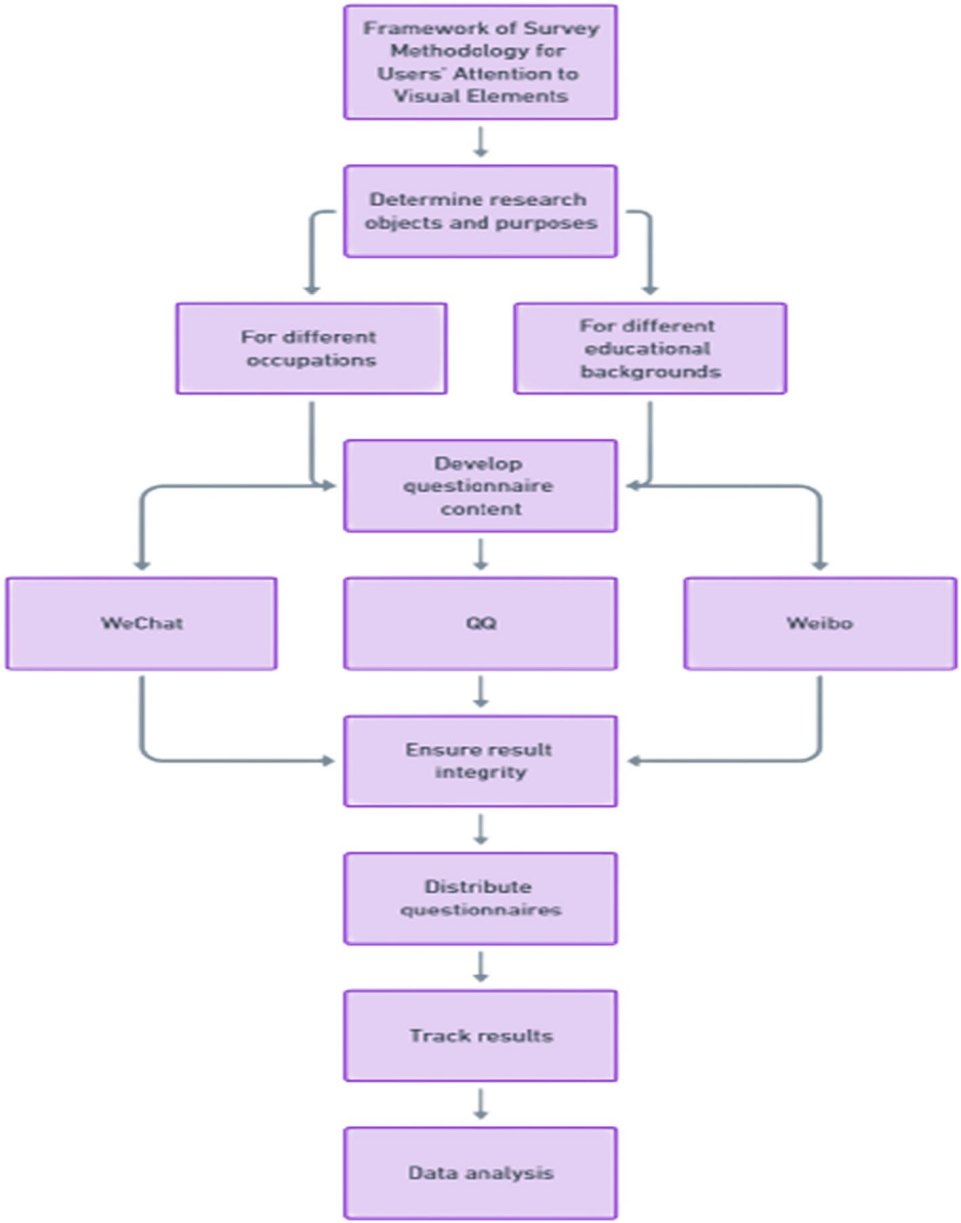
Survey methodology flowchart for users' attention to visual elements.

To effectively investigate users' attention to visual elements, a comprehensive survey methodology was designed, as shown in Fig. 1 in the flowchart below.

The flowchart covers the key steps of the survey methodology, including determining research objectives, developing questionnaires, distributing surveys, tracking responses, and analyzing data. This structured approach ensures that the user survey is conducted in a systematic and scientific manner.

Having established the need to analyze users' attention to visual elements, the next step is to design an effective methodology. This includes determining the survey objects, content, distribution methods and analysis approach. The goal is to gain insights into target users' needs and preferences, in order to guide the application of visual elements in web design.

### Ethics approval

The preliminary survey of this study involved issuing 200 questionnaires to investigate the degree of attention to visual elements in web pages among users of different occupations and educational backgrounds. Informed consent was obtained from all participants who took part in the questionnaire. This consent process was in compliance with the ethical standards of the Institutional Review Board (IRB) of the Affiliated Hospital of Guangdong Medical University. The IRB granted an ethics waiver for this study due to its non-interventional and observational nature, aligning with national regulations that exempt such studies from the requirement of formal ethics approval. Throughout the research process, we adhered to the ethical principles of the Declaration of Helsinki.

### Survey content and objects of user experience

Web design is to convey the information that the designer wants to transmit to users through the internet. In order to determine the needs of the viewers for the visual elements of the web page, sort out the main problems related to the visual elements in the web page design process, and use the questionnaire to investigate the current situation of the user's visual elements on the web page. According to the results of the questionnaire, determine the needs of different users for web visual elements. The subjects of the survey are mainly people who frequently search for information on the web. They can be users of different occupations and different educational backgrounds, but it is necessary to ensure a balanced ratio of men and women, and a total of 200 questionnaires were issued.

### Survey methods of users' attention to visual elements in web pages

For our user experience study, we disseminated 200 questionnaires across various online platforms, including We-Chat, QQ, and Weibo. Under the premise that the ratio of men to women is basically the same, samples are drawn according to users of different occupations and different educational backgrounds, and guarantees the completion of the survey results. In the survey of the user experience of visual elements in the web design process, we should first determine the survey object and survey purpose of this survey, and formulate the content of the questionnaire based on the content. After the questionnaire for the experience of visual elements in the web page is created, the designated personnel will release the questionnaire on different network platforms, and track the management of the survey results, and finally analyze the results of the questionnaire.

To delve deeper into the analysis of the attention given to visual elements by users of different educational backgrounds, we decided to employ a mathematical approach to quantify these data.

3 $${W}_{avg}=\frac{{\sum }_{i=1}^{n}{w}_{i}\times {a}_{i}}{{\sum }_{i=1}^{n}{w}_{i}}$$

where ${W}_{avg}$ represents the weighted average attention. $n$ denotes the number of categories based on different educational backgrounds. ${w}_{i}$ stands for the number of users (or weight) with the ${i}$th educational background. ${a}_{i}$ signifies the average attention to visual elements by users with the ${i}^{th}$ educational background. Utilizing the aforementioned formula, we conducted a weighted average analysis on the collected data.

### Educational applications of study findings

The findings of this study have a broad application in the field of education. For instance, educators can adjust their classroom teaching strategies based on our results, utilizing effective visual elements to enhance student interest and the appeal of instructional content. Educational institutions can also apply these strategies in their website design to better showcase educational resources, thereby attracting students and enhancing their learning experience.

In the following sections, we will detail the results of this analysis and discuss its implications for the practical application of visual elements in web design.

### Analysis of user needs for web visual elements

The characteristics of different web browsers are different. There are individual differences between them. The occupation, education level of the browser, what they see and hear in life, etc. It will affect the experience of the browser on the web-page. According to the suggestions provided by the viewer to the web designer during the process of browsing the web, the web designer makes the web content according to the actual needs of the user, let users have a better sense of use when browsing web pages, thereby improving the efficiency of web pages transmitting information to users. Before web design, on the premise of ensuring the ease of use of the web page, web designers should figure out the needs of target users, the content of the web-page should attract users in a short period of time, conform to the user's browsing habits, meet the needs of users through network visual elements, and convert the target users of the web-page into real users.

## Results and discussion

The analysis of the survey results regarding different users' attention to visual elements provides valuable insights for web design. However, simply analyzing user preferences is not enough. To further explore the application of visual elements in web design, it is also crucial to examine the effectiveness of visual information transmission.

We acknowledge that the sample of 200 self-recruited anonymous Internet users used in this study may not be representative of the entire Internet user population. As such, the conclusions of this study should be interpreted with caution and considered as preliminary insights into specific user behaviors. Future research should aim to include a broader sample to increase the generalizability of the findings.

### Analysis of the degree of attention of different professional users to visual elements

Users of different occupations may have different demands for browsing information online due to occupational reasons. According to the 200 questionnaires issued, the results shown in Table 1 can be obtained, according to Table 1, in this survey, 82 corporate staff paid the most attention to visual elements, and 2 social welfare workers were the least.

**Table 1:** Statistics on the attention of different professional users to visual elements.

Professional	Visual element attention
Students	34
Public institution personnel	18
Medical staff	29
Educator	11
Enterprise staff	82
Media worker	8
Social welfare worker	2
Other	16

**Figure 2:**
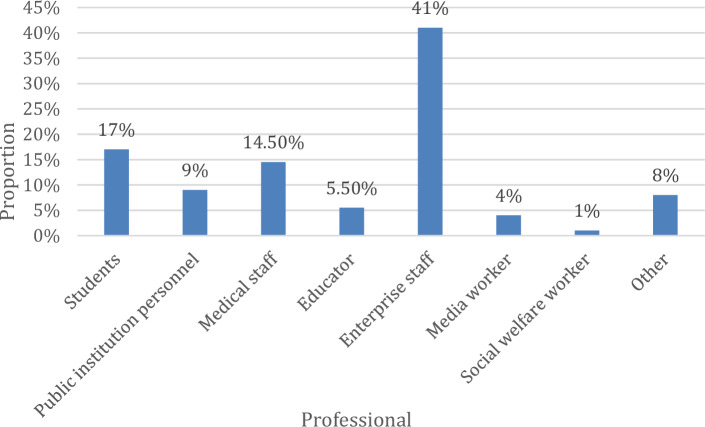
The proportion of different professional users' attention to visual elements.

According to the results in Table 1, the percentages of different professional users' attention to visual elements are shown in Fig. 2. From the figure, it can be seen that corporate employees' attention to visual elements accounts for the highest proportion of 41%, followed by students at 17%. Web designers can design career-related web interfaces according to different professional needs and their preferences for visual elements, for example, corporate workers pay more attention to text visual elements, while student groups pay more attention to video visual elements, and design according to user needs to achieve the effect of attracting users to browse the web for a second time. Web designers can combine the personality characteristics of young people and their hot spots to explore potential web browsing groups.

### Discussion on the variation of attention to visual elements among users of different educational backgrounds

Web content display varies when accessed by users from diverse educational backgrounds. This variation is often driven by backend algorithms that tailor content recommendations based on user browsing behavior. Our survey, encompassing 200 respondents, revealed distinct patterns in attention to visual elements. For instance, undergraduates, numbering 72, exhibited the highest attention to these elements. In contrast, individuals with doctoral degrees, totaling only 8, demonstrated the least attention. Furthermore, the collective attention from individuals with an educational level below a bachelor's degree was observed to be significant, with 71 respondents.

**Figure 3:**
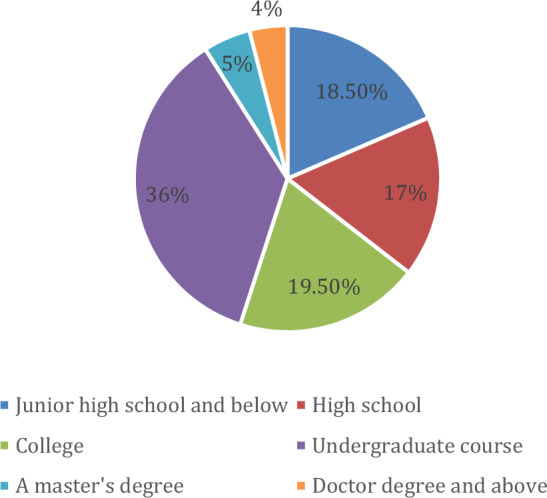
The proportion of users with different educational backgrounds paying attention to visual elements.

According to the results in Table 2, the proportion of users with different educational backgrounds' attention to visual elements is shown in Fig. 3. It can be seen from the figure that undergraduates' attention to visual elements accounts for 36% of the total proportion, and graduate students and doctoral degrees and above account for 36% of the total the ratio is less, 5% and 4% respectively. According to the analysis of education level, those with a high school degree or above accounted for 81.5% of the total surveyed people. Therefore, web designers need to consider the educational level of potential viewers and the visual element applications that different education levels like in the process of designing web pages. The text visual elements of the design need to conform to the user’s cultural background.

**Table 2:** Statistics on the attention of users with different educational backgrounds to visual elements.

Education level	Visual element attention
Junior high school and below	37
High school	34
College	39
Undergraduate course	72
A master's degree	10
Doctor degree and above	8

### Analysis of the effectiveness of visual information transmission in web design

The purpose of web design is to deliver web content to viewers. If there are no visitors to browse the web, then web information cannot be disseminated. Web design must first meet the psychological needs of viewers, and secondly, it must be able to attract users’ attention and reduce the risk of dissemination. Necessary information interferes with users to avoid unnecessary negative effects. The effectiveness of visual information transmission in web design is analyzed based on the following aspects:

1. Text and color elements: text is the most important part of transmitting web content, and color is the first visual language for human eyes to receive information. In web design art and cultural connotations should be combined in the process.
2. Images and panel elements: The main function of images is to convey information. In the web design process, images should be put in an appropriate amount. Too much will affect the effect of the entire web page to convey information. The panel can be carried out according to the designer's ideas in the web design process. To adjust, designers need to capture the public's aesthetics and design web pages.
3. Navigation bar: Setting a navigation bar on a web page can facilitate users to find target information. The location of the navigation bar is very important and requires careful consideration.

### Study limitations

There are several limitations to our study. Firstly, the public dataset used may not fully represent the behavior patterns of all Internet users. Secondly, the cross-sectional nature of the data limits our capacity for causal inferences. Lastly, we acknowledge the need for a more diverse sample to enhance the generalizability of our results.

## Conclusion

The burgeoning social and internet economy has witnessed a surge in the number of internet users globally. The integration of big data with corporate information has opened new avenues for personalized technology, especially in corporate web page design. Presently, web design exhibits a high degree of diversity, reflecting varied modes of information and emotional conveyance.

Given the diversity of internet users, understanding the aesthetic and browsing needs of the target audience has become paramount. Designers are required to conduct market research to cater to the preferences of different user groups. Visual elements play a pivotal role in this process. Appropriate color combinations, image, and graphic selections can enhance user experience, but it's essential to avoid overuse or misuse to ensure users aren't distracted or confused. The ubiquity of mobile devices also mandates designers to ensure that visual elements display correctly across various devices.

In summary, the significance of visual elements in web design cannot be overlooked. Designers must be adept at applying visual elements to create interfaces that align with public aesthetics while satisfying user browsing needs, thereby driving advancements in web page design.

To further guide web designers, we offer the following specific recommendations: Firstly, designers should adjust the use of visual elements based on the educational background of users, ensuring that the design aligns with the users' cultural and aesthetic habits. Secondly, considering the ubiquity of mobile devices, designers should ensure that visual elements are displayed correctly across various devices. Lastly, future research could explore the application of visual elements in different web design styles and how these elements affect user behavior and satisfaction, thus providing data-backed support for innovative developments in web page design.

## Data Availability

The datasets used during the current study available from the corresponding author on request.
